# Novel recombinant aminoacylase from *Paraburkholderia monticola* capable of N-acyl-amino acid synthesis

**DOI:** 10.1007/s00253-023-12868-8

**Published:** 2024-01-10

**Authors:** Gerrit Haeger, Tristan Jolmes, Sven Oyen, Karl-Erich Jaeger, Johannes Bongaerts, Ulrich Schörken, Petra Siegert

**Affiliations:** 1 Institute of Nano- and Biotechnologies, Aachen University of Applied Sciences, 52428 Jülich, Germany; 2 Faculty of Applied Natural Sciences, TH Köln University of Applied Sciences-Leverkusen Campus, Leverkusen, Germany; 3 Institute of Molecular Enzyme Technology, Heinrich Heine University Düsseldorf, 52425 Jülich, Germany; 4 Institute of Bio- and Geosciences IBG-1: Biotechnology, Forschungszentrum Jülich GmbH, 52425 Jülich, Germany

**Keywords:** Biosurfactants, Acyl-amino acids, Acylation, Aminoacylase, Biocatalysis, Chaperone

## Abstract

**Abstract:**

N-Acyl-amino acids can act as mild biobased surfactants, which are used, e.g., in baby shampoos. However, their chemical synthesis needs acyl chlorides and does not meet sustainability criteria. Thus, the identification of biocatalysts to develop greener synthesis routes is desirable. We describe a novel aminoacylase from *Paraburkholderia monticola* DSM 100849 (PmAcy) which was identified, cloned, and evaluated for its N-acyl-amino acid synthesis potential. Soluble protein was obtained by expression in lactose autoinduction medium and co-expression of molecular chaperones GroEL/S. Strep-tag affinity purification enriched the enzyme 16-fold and yielded 15 mg pure enzyme from 100 mL of culture. Biochemical characterization revealed that PmAcy possesses beneficial traits for industrial application like high temperature and pH-stability. A heat activation of PmAcy was observed upon incubation at temperatures up to 80 °C. Hydrolytic activity of PmAcy was detected with several N-acyl-amino acids as substrates and exhibited the highest conversion rate of 773 U/mg with N-lauroyl-L-alanine at 75 °C. The enzyme preferred long-chain acyl-amino-acids and displayed hardly any activity with acetyl-amino acids. PmAcy was also capable of N-acyl-amino acid synthesis with good conversion rates. The best synthesis results were obtained with the cationic L-amino acids L-arginine and L-lysine as well as with L-leucine and L-phenylalanine. Exemplarily, L-phenylalanine was acylated with fatty acids of chain lengths from C8 to C18 with conversion rates of up to 75%. N-lauroyl-L-phenylalanine was purified by precipitation, and the structure of the reaction product was verified by LC–MS and NMR.

**Key points:**

*• A novel aminoacylase from Paraburkholderia monticola was cloned, expressed in E. coli and purified.*

*• The enzyme PmAcy exhibits exceptional temperature and pH stability and a broad substrate spectrum.*

*• Synthesis of acyl amino acids was achieved in good yields.*

**Supplementary Information:**

The online version contains supplementary material available at 10.1007/s00253-023-12868-8.

## Introduction

Amino acid-based surfactants, especially acyl-amino acids, are molecules of interest for use in cosmetic products. They act as especially mild detergents and carry little inflammatory potential. In addition, they possess desirable foaming properties, are biodegradable, and have low toxicity (Zhao et al. 2019). The fatty acyl residue mediates lipophilic properties, while the amino acid acts as a polar head group. Variances in both the amino acid and the fatty acid moieties can generate structural variability (Pinheiro and Faustino 2017). Exemplarily, mild surfactant properties are gained with, e.g., lauric acid (C12) or myristic acid (C14) as acyl groups, whereas longer acyl chains like stearic acid (C18) or oleic acid (C18:1) yield emulsifiers. Being a valuable compound in cosmetics, amino acid-based surfactants are remarkably skin-protective.

While acyl-amino acids are environmentally benign regarding application and biological degradation, the conventional chemical synthesis of these compounds via the Schotten-Baumann reaction is not sustainable (Ibrahim et al. 2020). Chlorinated fatty acids have to be used which are typically produced with toxic phosgene or thionyl chloride. During chlorination and acylation significant amounts of waste products like phosphoric or sulfuric acid and sodium chloride are produced. Additionally, not all acyl-amino acids can be synthesized with the same efficiency. Secondary amino groups have to be protected in order to prevent side reactions (Joondan et al. 2018). Hence, a demand exists to replace the chemical production with a biocatalytic synthesis approach.

Aminoacylases (EC 3.5.1.14) are hydrolytic metallo-enzymes that act on amide bonds of N-acyl-L-amino acids. Enzymes belonging to this diverse class have been described from several organisms. The natural function of these enzymes remains largely unknown, but for mammalian aminoacylases, functions in catabolism of acetyl-peptides or acetyl-amino acids or xenobiotic detoxification have been described (Anders and Dekant 1994). Nonetheless, aminoacylases are enzymes for technical applications, for both hydrolysis and synthesis of N-acyl-amino acids. First process implementations are found using fungal aminoacylases from *Aspergillus* sp. for the production of enantiomerically pure amino acids from racemically acetylated amino acids (Gentzen et al. 1980; Liu et al. 2006). The fungal enzyme has been evaluated for acylation of amino acids; however, yields were below 5% and thus unsatisfactory for commercial application (Kimura et al. 1992). Among all known aminoacylases, the mammalian enzyme pAcy1 from porcine kidney has been described in most detail. The hydrolytic reaction mechanism, in context with dimerization of the enzyme, has been postulated (Lindner et al. 2005; Liu et al. 2006). By directed mutagenesis, the synthesis to hydrolysis-ratio could be altered (Wardenga et al. 2010). With the addition of glycerol to the reaction mixture, yields could be improved, but product concentration remained low (< 5 mM) as an excess of amino acid with a low fatty acid concentration was employed (Wada et al. 2002). The stability of the (hog) acylase is poor under favorable synthesis conditions (Henseling and Röhm 1988). In addition, the heterologous expression of soluble, affinity-tagged pAcy1 in *E. coli* has been proven difficult, as the protein is prone to aggregation (Wardenga et al. 2008).

Several bacterial aminoacylases from streptomycetal origin that are capable of N-acyl-amino acid synthesis have been published. Initially, four aminoacylases from *S.* *mobaraensis* have been investigated. An epsilon-lysine-acylase (SmELA) that synthesizes N_ε_-lauroyl-lysine (Koreishi et al. 2009a) and an extracellular, multimeric penicillin V acylase (SmPVA; EC 3.5.1.11) capable of synthesis of several lauroyl-amino acids have been described. Also, a broad-spectrum alpha-aminoacylase (SmAA) was cloned and characterized, but no synthesis has been shown. Those enzymes were heterologously expressed in *S. lividans* (Zhang et al. 2007; Koreishi et al. 2009b). Synthetic activity towards acyl-amino acids has also been shown from cell extract of *S. ambofaciens*. The activity could be attributed to homologs of the *S. mobaraensis* enzymes, mainly to SamAA (Bourkaib et al. 2020a; Dettori et al. 2018). Furthermore, homologs of SmPVA were found in *S. lavendulae* (Torres-Bacete et al. 2015) and *Streptomyces* sp. No. 6907 (Ueda et al. 2011). None of the streptomycetal enzymes have been expressed in *E. coli* yet. The aminoacylase MsAA from *Mycolicibacterium smegmatis*, which is homologous to SamAA, has been heterologously expressed in *E. coli* and *V. natriegens* and is capable of lauroyl-amino acid synthesis (Haeger et al. 2023).

Recently, an aminoacylase from *Burkholderia* sp. was characterized that can produce several N-lauroyl-L-amino acids to high yields in aqueous buffers, reaching 51% for N-lauroyl-L-phenylalanine and even 89% for N-lauroyl-L-arginine due to product precipitation (Takakura and Asano 2019). The enzyme was remarkably stable at high temperatures and broad pH values and in the presence of inhibitors and chelators, which is an important trait for production processes. As with the streptomycetal aminoacylases, there was no need for enzyme immobilization or the use of organic solvent to achieve competitive yields. The authors found close homology of the enzyme only to several uncharacterized hydratases and low homology (≤ 36%) with several proteins belonging to the metal-dependent hydrolase superfamily from various microorganisms. The aminoacylase was attempted to be heterologously produced in *E. coli* BL21 (DE3); however, only traces of recombinant aminoacylase activity could be detected.

Since the enzyme from *Burkholderia* sp. possesses highly interesting traits for industrial biocatalytic production of N-lauroyl-L-amino acids but is hampered in heterologous expression, the search for homologs of the enzyme that can be produced in *E. coli* seems worthwhile. In our study, we cloned an aminoacylase gene from *Paraburkholderia monticola* DSM 100849, designated PmAcy, for heterologous expression in *E. coli* BL21 (DE3). After purification to homogeneity by affinity chromatography, the enzyme was subsequently biochemically characterized and evaluated for synthesis of acyl-amino acids.

## Materials and methods

### Chemicals and reagents

Unless stated otherwise, chemicals were analytical grade and purchased from Sigma-Aldrich (USA). Amino acids, culture media components, NaCl, and Tris (tris(hydroxymethyl)aminomethane) were from Carl Roth (Germany), and acetyl-amino acids were either from Sigma or Bachem (Switzerland). BSA (bovine serum albumin, fraction V) was from PanReac AppliChem (Germany).* N*-Lauroyl-, *N*-palmitoyl-, and other *N-*acyl-amino acids were synthesized by the Schotten-Baumann-reaction as previously described (Haeger et al. 2023). Reagents for molecular biology were from Thermo Fisher Scientific (USA). Gene synthesis was ordered from GeneArt service by Thermo Fisher Scientific. DNA oligonucleotide synthesis and DNA sequencing were performed by Eurofins Genomics (Germany). Strep-Tactin®⁠ columns were from IBA (Germany). The EZ Nin reagent (Biochrom, UK) was used for amino acid quantification.

### Bacterial strains and plasmids

*E. coli* DH5α (F^–^ φ80*lac*ZΔM15 Δ(*lac*ZYA-*arg*F)U169 *rec*A1 *end*A1 *hsd*R17(r_K_^–^, m_K_^+^) *pho*A *sup*E44 λ^–^ *thi*-1 *gyr*A96 *rel*A1) was used for cloning. *E. coli* BL21 (F^–^ *ompT gal dcm lon hsdS*_*B*_(*r*_*B*_^–^*m*_*B*_^–^) λ(DE3 [*lacI lacUV5*-*T7p07 ind1 sam7 nin5*]) [*malB*^+^]_K-12_(λ^S^)) was used for protein expression. As expression plasmid, pET28a-based plasmid with recombinant aminoacylase genes was used. The resulting strains were *E. coli* BL21 (DE3) pET28a PmAcy NTag/CTag/noTag. The plasmid pGro7 (Takara Bio Inc.) was used for co-expression of chaperones. Hence, *E. coli* BL21 (DE3) pGro7 pET28a PmAcy NTag was used for recombinant aminoacylase production. The N-terminally tagged enzyme is referred to as PmAcy.

### Sequence analysis and cloning of the aminoacylase gene from *Paraburkholderia monticola*

Homology searches were performed by the program BLASTp provided by NCBI (Altschul et al. 1990). The gene accession number in the NCBI database is KXU85199.1. Multiple sequence alignment was conducted with Clustal Omega (https://www.ebi.ac.uk/Tools/msa/clustalo/) and displayed with ESPript (https://espript.ibcp.fr/ESPript/ESPript/). Upon adding both N-and C-terminal StrepII-Tag sequences with additional serine-glycine-linkers, the protein sequence was reverse translated based on *E. coli* codon usage. The DNA string was ordered as a GeneArt String from Thermo Fisher. The DNA string was amplified with primers carrying BsaI overhangs for golden gate cloning (Table S1). The variants of the gene were cloned, either without affinity tag (noTag, amplified with primer P2 and P4) or with an N- or C-terminal StrepII-tag (NTag or CTag, amplified with primers P1 and P4 or P2 and P3, respectively). The genes were cloned into pET28-eforRED by golden gate assembly using BsaI. Transformation of *E. coli* cells was performed with the heat shock method (Hanahan 1983). The DNA and protein sequences of PmAcy, along with the primer sequences, are included in the supplemental material.

### Production of recombinant aminoacylase in *E. coli* and purification

For protein expression *E. coli* BL21 (DE3) pGro7 pET28a PmAcy (NTag, CTag, or no tag) was grown in Terrific Broth-autoinduction medium (TB-AIM; 2% tryptone from casein, 2.4% yeast extract, 25 mM NaH_2_PO_4_, 25 mM KH_2_PO_4_, 50 mM NH_4_Cl, 2 mM MgSO_4_, 5 mM Na_2_SO_4_, 0.5% glycerol (v/v), 0.5% lactose, and 0.05% glucose). Cells were cultivated for 24 h in autoinduction media. Kanamycin was added at a concentration of 50 µg mL^−1^. For co-expression of chaperones from pGro7, 25 µg mL^−1^ chloramphenicol and 0.5 mg mL^−1^ arabinose were added. A 10-mL preculture was grown at 180 rpm and 37 °C overnight. In a 1-L flask, 100 mL of expression medium was inoculated with the preculture to a final OD_600_ of 0.2 and grown at 20 °C with shaking at 225 rpm. The cells were harvested by centrifugation at 3000 g and 4 °C for 40 min.

Cells were disrupted by sonication with a sonotrode (Bandelin). The lysis buffer consisted of 100 mM Tris–HCl pH 8.0 supplemented with 0.1% Triton X-100, 1 mM ZnCl_2_, 0.3 mg mL^−1^ lysozyme, and 150 mM NaCl. Per gram of harvested cells, 10 mL of lysis buffer was added to the cell pellet. Sonication was conducted for 3 × 4 min at 50% power and 50% cycle. After lysis, the samples were centrifuged at 16,000 g and 4 °C for 40 min. The insoluble fraction was resuspended in 8 M urea.

Recombinant aminoacylases were purified by Strep-tag affinity chromatography using a Strep-Tactin® SuperFlow® high capacity cartridge (IBA) according to the manufacturer’s instructions. The buffer used for purification was 100 mM Tris–HCl pH 8.0, 1 mM ZnCl_2_, and 150 mM NaCl and contained 2.5 mM desthiobiotin for elution of PmAcy. The fractions containing the recombinant enzyme were pooled and washed with elution buffer using Vivaspin 6 concentrators (10,000 MWCO; Sartorius) to remove desthiobiotin.

### Determination of protein concentration, purity, and molecular mass

Protein concentrations were determined with the Bradford method (Bradford 1976) using the Roti®-Nanoquant reagent (Carl Roth). Bovine serum albumin (BSA) served as a standard.

The SDS-PAGE analysis of proteins was conducted according to Laemmli (Laemmli 1970) using 8–20% gradient polyacrylamide gels and staining with Roti®Blue quick (Carl Roth). As a protein ladder, FastGene® BlueEasy Protein Marker (Nippon Genetics) was used.

To determine the native molecular weight, blue native PAGE was performed using reagents from SERVA (Germany) and conducted according to the manufacture’s protocols using SERVAGel™ N4-16% gels.

Matrix-assisted laser desorption ionization-time of flight mass spectrometry (MALDI-TOF/MS) analysis was conducted to determine the molecular mass of the monomeric enzyme. The Axima Confidence device (Shimadzu Europe, Duisburg, Germany) was used in linear positive mode with pulsed extraction optimized for the theoretical molecular weight. Data analysis was conducted with mMass (Strohalm et al. 2008). Concentration of the protein solutions was 1 mg/mL. The samples were diluted tenfold with α-cyano-4-hydroxycinnamic acid (CHCA) (Sigma-Aldrich, USA). On each target plate spot, 2 μL of the sample were applied. Trypsinogen and BSA from LaserBio Labs were used as molecular weight standards.

### Aminoacylase activity assay

Activity of aminoacylases was determined as previously described (Haeger et al. 2022). From 200 μL aminoacylase reactions, 10 μL samples were mixed with 100 μL of EZ Nin:DMSO reagent and heated for 10 min at 99 °C. Afterwards, the samples were diluted 1:10 with 100 mM Na-borate buffer pH 10.0 for measurement in microtiter plates. The reactions consisted of 190 μL substrate solution and 10 μL enzyme solution. For standard hydrolytic activity measurements, reactions with 15 mM N-lauroyl-L-alanine in 100 mM Na-borate buffer pH 9.0 were performed at 30 °C for 5 min. If not stated otherwise, ZnCl_2_ concentration in the reaction was 50 µM. One unit of PmAcy was defined as the amount of enzyme that hydrolyzes one µmol of N-lauroyl-L-alanine per minute under the given conditions. For determination of kinetic parameters, volumes of the ninhydrin assay were adjusted to increase sensitivity at low substrate concentrations, so that 50 μL sample was mixed with 200 μL EZ Nin:DMSO. Then, 200 μL of the colored sample was mixed with 50 μL 100 mM Na-borate pH 10.0.

### Biochemical characterization of PmAcy

The optimal pH of the hydrolytic reaction was investigated by using following buffers: Na-citrate for pH 5.0, Na-MES (2-(N-morpholino)ethanesulfonic acid) for pH 6.0 and 7.0, Tris–HCl for pH 6.0–9.0, and Na-borate for pH 9.0–12.5. Due to poor solubility at low pH values, only 3 mM N-lauroyl-L-alanine was prepared in 50 mM of buffer and adjusted to the respective pH. The purified enzyme solution was diluted 1:10 in each buffer to a final protein concentration of 2 µg/mL. The reactions were conducted for 10 min at 30 °C with sampling each minute and analysis of initial reaction velocity.

The pH stability was assessed in 100 mM of Na-acetate buffer at pH 4.0–6.0, Na-MES buffer at pH 5.0–7.0, Tris–HCl at pH 6.0–9.0, and Na-borate buffer at pH 9.0–13.0 at 30 °C without agitation. From the incubation solutions containing 240 µg/mL enzyme, 10 μL was withdrawn and used for activity measurements after 1 h and 24 h. As a substrate, 15 mM N-lauroyl-L-alanine was used in 200 mM Na-borate pH 9.0. The borate buffer concentration in the assay was altered compared to standard conditions to prevent a significant pH shift caused by the incubation buffers upon addition of the incubated enzyme. The reaction was conducted at 30 °C. Final enzyme and ZnCl_2_ concentrations were 12 µg/mL and 10 µM, respectively.

The optimal temperature for hydrolysis was determined by measuring the activity in a range of 20–90 °C. Reactions were conducted with 15 mM N-lauroyl-L-alanine in 100 mM Na-borate pH 9.0 and 1–4 µg/mL purified enzyme.

For the assessment of temperature stability or thermal activation, purified PmAcy was incubated at temperatures from 20 to 90 °C in 100 mM Tris–HCl buffer at pH 8 and residual activity was determined after 1 h, 24 h, and 4 days. Reactions were carried out at a PmAcy concentration of 60 µg/mL in a volume of 200 μL. Residual activity was determined with the standard aminoacylase assay (15 mM N-lauroyl-L-alanine in 100 mM Na-borate buffer pH 9.0 at 30 °C).

### Effect of metal ions and substrate specificity

The effect of various bivalent metal ions on the hydrolytic activity of PmAcy was investigated by incubating 1 mg/mL of the purified enzyme (no ZnCl_2_ added to the buffer) with various ions at a final concentration of 0.1 nM to 1 mM (CaCl_2_, CoCl_2_, CuCl_2_, FeSO_4_, MgCl_2_, MnCl_2_, NiCl_2_, and ZnCl_2_) for 1 h at room temperature. After the incubation period, activity was measured with the standard assay. In the absence of divalent metal ions, no activity could be measured. The activity determined by addition of zinc was defined as 100%.

The substrate scope was determined by hydrolysis of various substrates at 15 mM in 100 mM Na-borate, pH 9.0 at 50 °C. The reaction temperature was higher than the standard conditions to solubilize all substrates, because N-palmitoyl-L-amino acids and N-lauroyl-L-cysteine were not soluble at 30 °C. The concentrations of purified enzyme were 6–60 µg/mL. The final concentration of bivalent metals (ZnCl_2_, CoCl_2_, or MnCl_2_) in the reactions was 50 µM. The activity was determined from the initial reaction velocity.

To determine *K*_*M*_ and *V*_*max*_ of the hydrolysis reaction towards N-lauroyl-L-alanine and N-lauroyl-L-phenylalanine as substrates, a concentration range of 50 µM to 15 mM of the substrates was prepared in 100 mM Na-borate pH 9.0 at 50 °C. The enzyme concentration was 1.2 µg/mL, and initial reaction velocities were analyzed in sampling intervals of 30 s. The kinetic parameters were determined with GraphPad Prism 8 software.

### Thermal shift assay

Thermal shift assay was used as previously described (Falkenberg et al. 2022) to investigate thermal denaturation of PmAcy in real time. After mixing the protein with the fluorogenic dye SYPRO® orange, the sample was heated stepwise. An increase in fluorescence during the heating procedure indicates a denaturation of the protein due to exposed hydrophobic areas. 10 μL protein samples (> 0.1 mg/mL) are mixed with 5 μL 50 × SYPRO Orange (Sigma Aldrich) and 20 μL 10 mM HEPES (4-(2-hydroxyethyl)-1-piperazineethanesulfonic acid) pH 8.0. 10 mg/mL lysozyme (Serva) served as a positive control. Measurement was performed with qTower3G and qPCRsoft 4.0 (Analytik Jena) using the TAMRA Channel (*λ*_*ex*_ = 535 nm, *λ*_*em*_ = 580 nm). The heating program was 25 °C to 95 °C with steps of 2 °C, 120 s hold time per temperature and a heating speed of 4.4 °C/s.

### Biocatalytic synthesis of N-acyl-amino acids

In general, the reaction mixtures consisted of 200 mM amino acid, 100 mM fatty acid, 500 µM ZnCl_2_ in 50 mM buffer Tris–HCl buffer pH 8.0 in a volume of 1 mL. The reactions were started by addition of 0.6 Units aminoacylase PmAcy (standard activity assay), and the syntheses were run at 50 °C and 250 rpm for 24 h in 25 mL vessels on an *Infors TM* orbital shaker. Fatty acids, amino acids, and pH were varied in a “one factor at a time” (OFAT) approach. The pH was varied from 7.0 to 13.0 using either 50 mM Tris–HCl buffer (pH 6–9) or 50 mM borate buffer (pH 9.0–13.0). Different fatty acids from C8 to C18 were analyzed with L-phenylalanine as amino acid cosubstrate. For carbon chain length from C16 to C18, 10% (v/v) ethanol was added to the reaction mixture for solubilization purposes. All 20 natural amino acids were tested in the presence of lauric acid as cosubstrate. The reactions were stopped by addition of 990 μL acetonitrile and incubation at 100 °C for 30 min and analyzed by HPLC-ELSD and LC–MS. N-lauroyl-L-phenylalanine was synthesized in 5-mL scale and purified exemplarily according to the method described in the “Scale-up and isolation of N-lauroyl-L-phenylalanine” section.

### Scale-up and isolation of N-lauroyl-L-phenylalanine

200 mM L-phenylalanine, 100 mM sodium laurate, 0.5 mM ZnCl_2_, and 3.0 Units PmAcy (standard activity assay; 60 µg) were dissolved in 50 mM Tris-buffer pH 8.0 at a total volume of 5 mL. The synthesis reactions were conducted at 50 °C and 250 rpm for 1 h. Afterwards, the pH-value of the reaction mixture was set to 1 by addition of 5 N hydrochloric acid. The precipitate was separated from the liquid phase by centrifugation, dried *in vacuo*, and analyzed.

### Chromatographic and spectroscopic product analysis

The conversion of amino acids to *N-*lauroyl-L-amino acids was monitored by HPLC-ELSD with a Shimadzu Nexera XR system equipped with a Hitachi LaChrom II column (C18, 5 µm, 4.6 × 250 mm) and an ELSD 100 (evaporative light scattering detector) from VWR. The products were dissolved in an equal volumetric mixture of acetonitrile and water, and 20 μL was injected to the column. The column temperature was set to 40 °C, and the products were separated in a gradient starting from starting from 80% water (supplemented with 0.1% formic acid):20% acetonitrile to 100% acetonitrile within 10 min. The concentration was held for 6 min, and the gradient was set back to 20% acetonitrile over the course of 2 min. The flow rate was set to 1 mL/min. Amino acids and acyl-amino acid concentrations were calibrated with 1 mM ethyl-L-tryptophanate hydrochloride (TrpOEt-HCl) solution as internal standard.

LC–MS analysis was conducted using a Shimadzu Nexera XR system equipped with a Hitachi LaChrom II column (C18, 5 µm, 4.6 × 250 mm) and a Shimadzu LCMS-2020-mass spectrometer. Applying the same analysis conditions as in HPLC-ELSD analysis allowed a direct comparison of the chromatograms.

NMR spectroscopy of N-lauroyl-L-phenylalanine was done with a Bruker Avance III HD 400 MHz system. 30 mg N-lauroyl-L-phenylalanine was dissolved in 650 μL CDCl_3_. Proton nuclear magnetic resonance (^1^H-NMR) spectra were analyzed at 400 MHz and chemical shifts are reported in *δ* units (ppm) with TMS as reference (*δ* 0.00). Carbon nuclear magnetic resonance (^13^C-NMR) spectra were measured at 100 100 MHz, and chemical shifts are reported in *δ* units (ppm) with CDCl_3_ as reference *δ* (77.00).

## Results

### Cloning of the *pmAcy* gene from *Paraburkholderia monticola* and expression in *E. coli*

The putative aminoacylase from *P. monticola* DSM 100849 is a homolog of the long-chain aminoacylase from *Burkholderia* sp. Strain LP5_18B (Takakura and Asano 2019) with 85.5% homology by sequence identity as identified via NCBI BLASTp algorithm (GenBank: KXU85199.1). The synthetic DNA fragment (GenBank OR188138) was cloned into the pET28a expression vector via Golden Gate cloning. The DNA string contained both N- and C-terminal StrepII-tag sequences. The gene was cloned either with N- or C-terminal Strep-tag or without an affinity tag. The resulting plasmids were designed as pET28a PmAcy (Ntag/Ctag). The theoretical molecular weight of the protein is 47.4 kDa (48.7 kDa with Strep-tag).

**Fig. 1:**
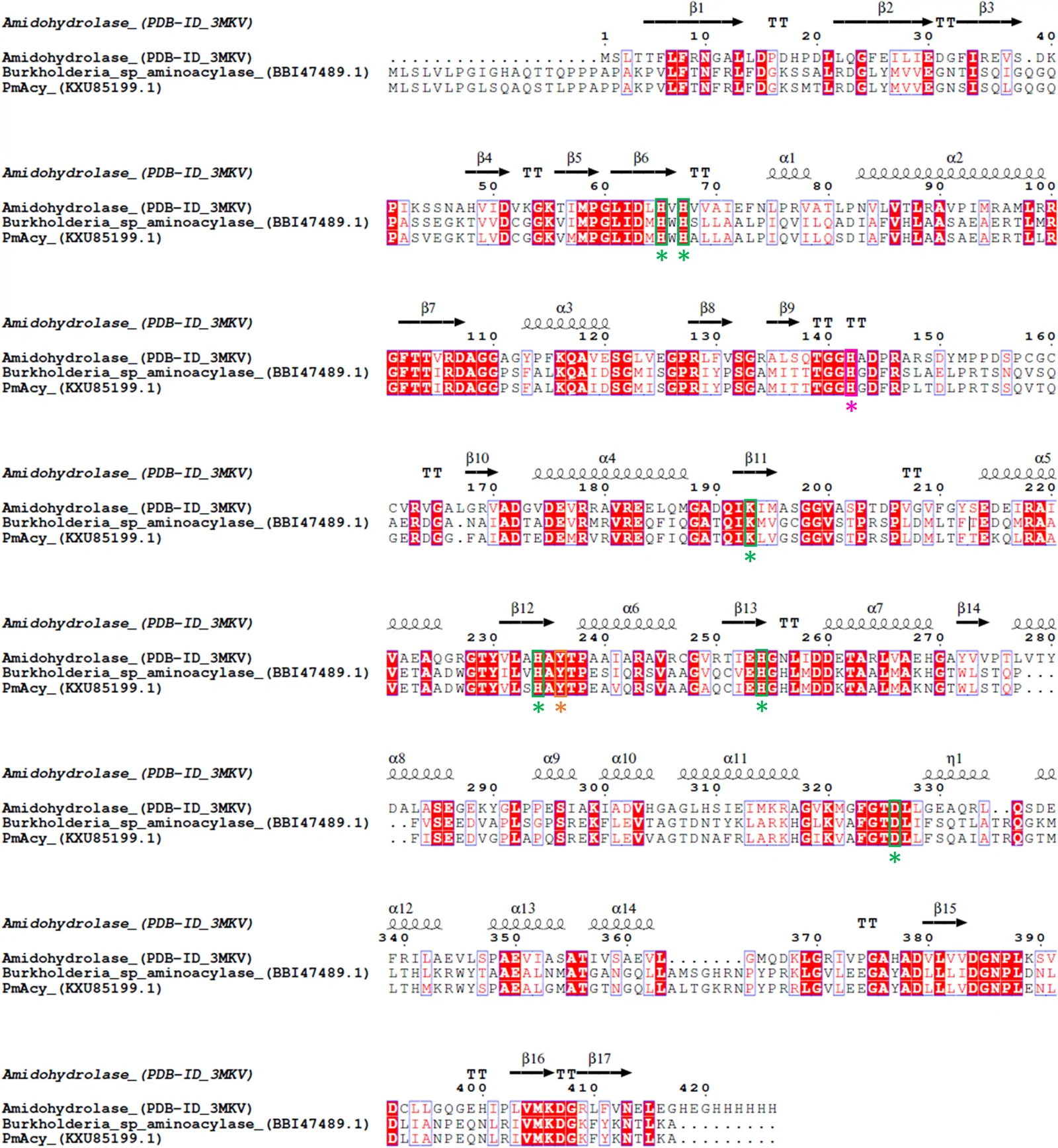
Protein sequence alignment. PmAcy (KXU85199.1), the aminoacylase from *Burkholderia* sp. strain LP5_18B, and an amidohydrolase (PDB: 3MKV) were aligned. The alignment was generated using the Clustal Omega tool and displayed with ESPript 3.0. The conserved metal-binding residues (four histidines, one aspartic acid, and one lysine residue) are highlighted by green boxes and asterisks. The conserved histidine described to be oxyanion-hole forming is highlighted in pink. The conserved tyrosine residue that binds the α-carboxylic group of amino acid substrates in amidohydrolases is highlighted in orange. The annotation of the secondary structure, based on the structure of 3MKV, is displayed as arrows for β-strands, squiggles for α-helixes, and the letters TT for turns

**Fig. 2:**
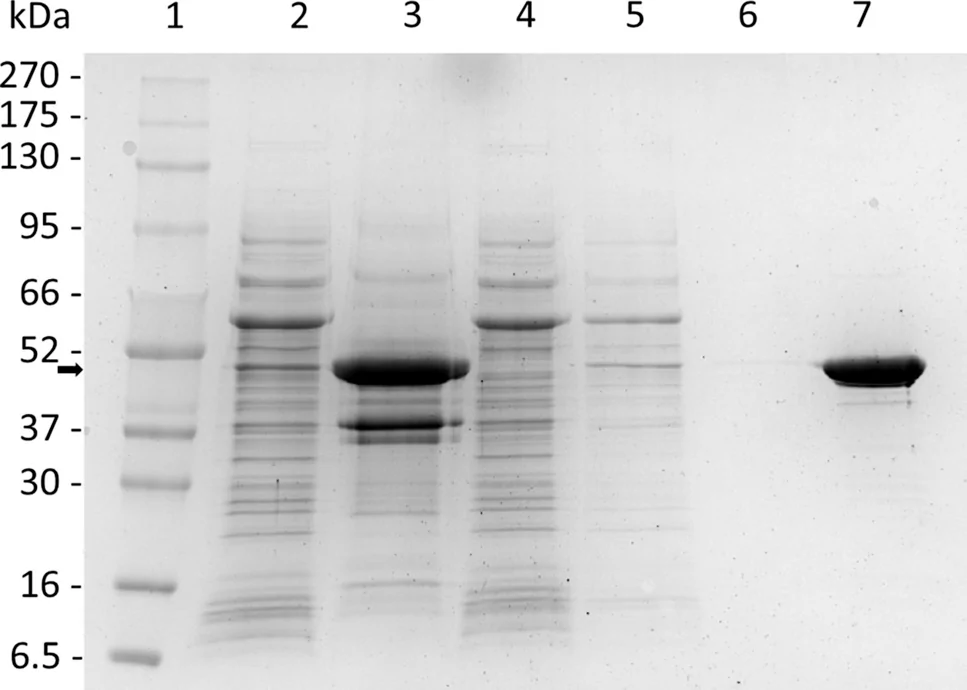
SDS-PAGE analysis of different fractions obtained during purification of PmAcy. The enzyme was purified from a culture of *E. coli* BL21 pGro7 + pET28a PmAcy (TB medium-autoinduction). Lane 1: protein marker (BlueEasy Prestained Protein Marker, Nippon Genetics); lane 2: cell-free extract; lane 3: insoluble fraction; lane 4: flow-through from Strep-tag affinity chromatography; lane 5: first wash fraction; lane 6: second wash fraction; lane 7: Elution of PmAcy. The black arrow indicates the migration height of PmAcy

Sequence analysis revealed that PmAcy belongs to the conserved protein domain family of metallo-dependent hydrolases (subgroup A). This group of proteins shows a conserved TIM barrel 3D structure (Lu et al. 2020) and can be classified a member of the M38 family from the MJ clan in MEROPS peptidase database classification. The characteristic metal-binding site comprising four histidine residues (H85/87/253/273) and one aspartic acid residue (D340) is conserved in the sequence of PmAcy. In a homologous amidohydrolase structure (PDB-ID: 3MKV; 33% identity), the zinc ions are bridged by a carboxylated lysine residue (Xiang et al. 2010), and the corresponding lysine residue is conserved in the sequence of PmAcy as well (K212). Other conserved residues include H162, which is described as an oxyanion-hole forming residue to stabilize the reaction intermediate, and Y255, which serves to bind the α-carboxylic group of the substrate (Xiang et al. 2010). A protein sequence alignment of PmAcy with the sequences of the *Burkholderia* aminoacylase (Takakura and Asano 2019) and the referenced amidohydrolase (3MKV) with highlighted active site residues is shown in (Figs. 1 and 2).

The aminoacylase PmAcy was prone to aggregation when heterologously produced in *E. coli* BL21 (DE3). The expression experiments using 1 mM IPTG for induction at 37 °C and 20 °C yielded only inclusion bodies. Lowering the inductor concentration to 0.1 mM IPTG at 20 °C did not yield soluble protein either. Furthermore, switching to autoinduction with lactose did not help to increase the solubility. By co-expression of the chaperonin GroEL/ES in *E. coli* BL21 (DE3) pGro7 at 20 °C using induction with lactose and arabinose, soluble and active protein could be obtained. Still, the majority of PmAcy was found in the insoluble fraction. In comparison, the N-terminally tagged variant of PmAcy was produced best (data not shown) and used for further purification and enzyme characterization.

### Purification of the N-terminally StrepII-tagged aminoacylase PmAcy

The enzyme was typically enriched 16-fold, and 93.8% of the enzyme was recovered with 2288 U, and 3.0 U/mg in the cell-free extract and 2146 U in the elution fractions. The specific hydrolytic activity of purified PmAcy Ntag was determined as 50 U/mg with the substrate N-lauroyl-L-alanine using a standard activity assay (30 °C reaction temperature, pH 9.0). From 100 mL expression culture, approximately 15 mg purified PmAcy were yielded. As Fig. 2 shows, the enzyme was purified to homogeneity with an apparent molecular mass of approximately 50 kDa. The mass of purified PmAcy NTag was verified by MALDI-TOF analysis to be 48.7 kDa, as expected from its similar theoretical molecular weight. Native PAGE revealed that the aminoacylase is a multimeric enzyme which migrates at a position of M_r_ 550 kDa, suggesting a dodecameric form (Figs S1, S2).

### Biochemical characterization of PmAcy

**Fig. 3:**
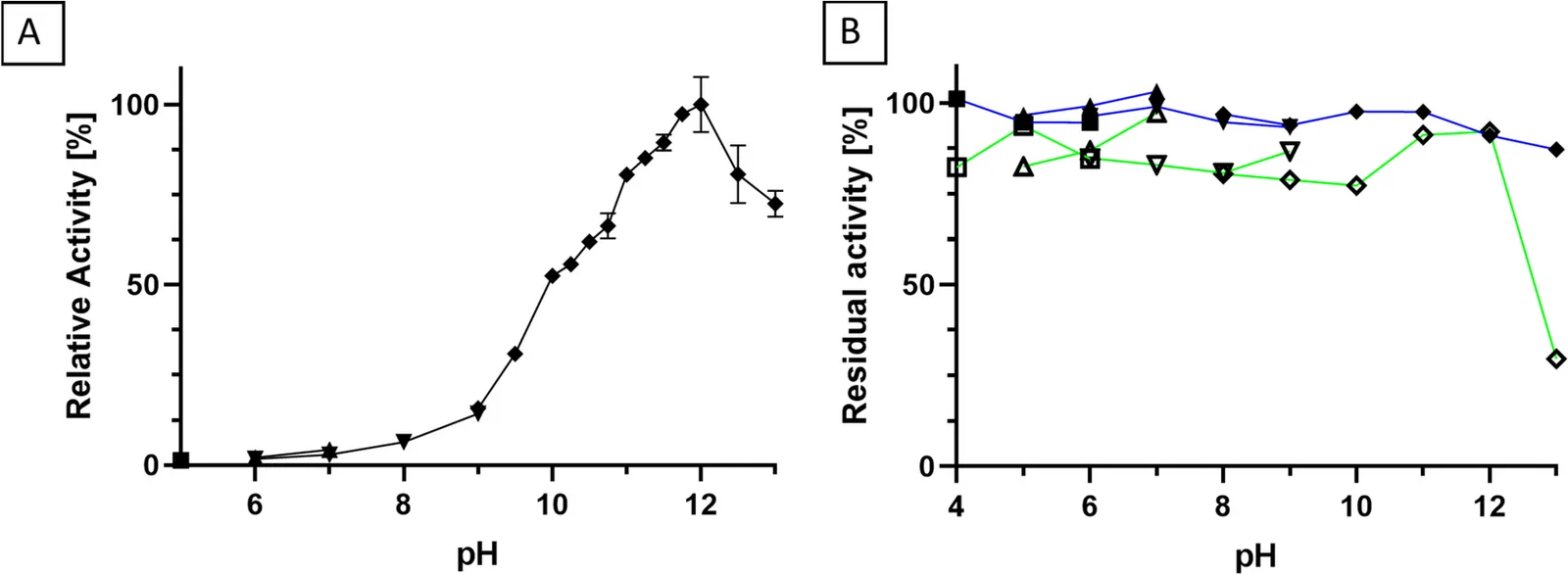
Influence of pH on activity and stability of PmAcy. **A** pH dependency of hydrolytic activity of PmAcy against N-lauroyl-L-alanine. Reaction conditions: 3 mM N-lauroyl-L-alanine at 30 °C reaction temperature. The following buffers were used at 50 mM: Na-citrate for pH 5.0 (●), Na-MES for pH 6.0 and 7.0 (▲), Tris–HCl for pH 6.0–9.0 (▼), and Na-borate for pH 9.0–13.0 (◆). All reactions were conducted in triplicates. **B** pH dependency of stability of PmAcy after 1 h and 24 h. The following buffers were used at 100 mM: Na-acetate for pH 4.0–6.0 (■/□), Na-MES for pH 5.0–7.0 (▲/△), Tris–HCl for pH 6.0–9.0 (▼/▽), and Na-borate for pH 9.0–13.0 (◆/◇). Activities measured after 1 h and 24 h are depicted with filled and empty symbols, connected by blue and green lines, respectively. Residual activity was determined with the standard assay conditions and indicated as percental values. All reactions were conducted in triplicate

The hydrolysis of N-lauroyl-L-alanine at 30 °C was used as a standard reaction for the biochemical characterization of purified PmAcy. To determine the pH optimum, hydrolytic activities were analyzed in various buffers ranging from pH 5–12.5 (Fig. 3A). Experiments at lower pH-values were not possible, because the substrate was insoluble. The highest activity of 278 U/mg was obtained at pH 12, which was set to 100% relative activity (Fig. 3A). Only weak activity was detected in the neutral to acidic pH range. The pH stability of PmAcy was assessed by incubating the enzyme at 30 °C for 1 h and 24 h in buffers ranging from pH 4–13 (Fig. 3B). After incubation, the remaining hydrolytic activity was measured by the standard activity assay (30 °C, pH 9.0). The enzyme was stable in all buffers for 1 h, and still > 80% activity remained at pH 4–12 after 24 h of incubation. Only at pH 13 significant deactivation was observed. Still, the enzyme is sufficiently stable at alkaline conditions, and 92% of its initial activity were retained after 24 h at the optimum pH of 12.

**Fig. 4:**
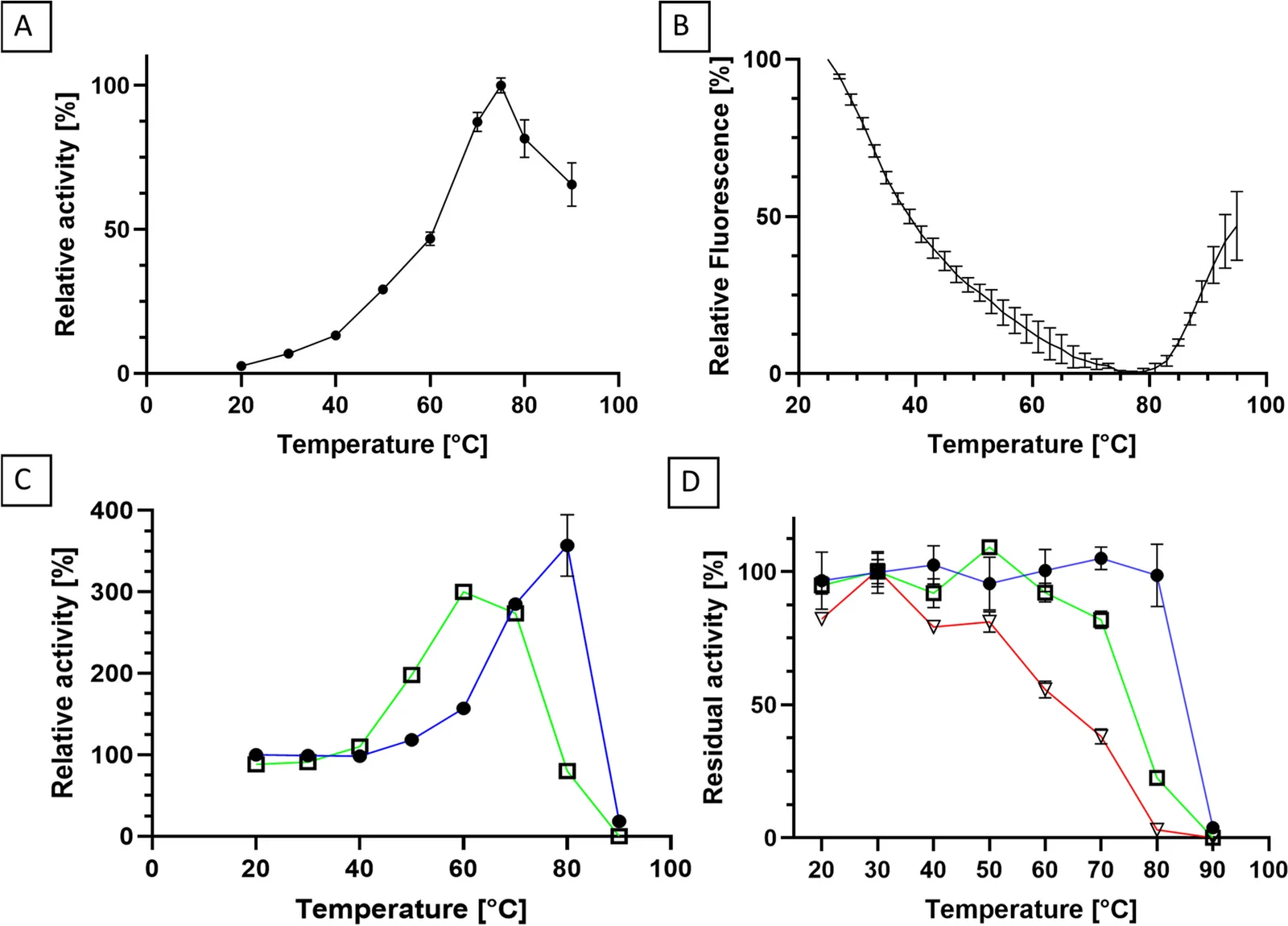
Influence of temperature on activity and stability. **A** Temperature dependency on hydrolytic activity against N-lauroyl-L-alanine. Reaction conditions were 15 mM N-lauroyl-L-alanine in 100 mM Na-borate pH 9.0 at various reaction temperatures. **B** Thermal shift assay with PmAcy. Thermal denaturation was followed via fluorescence measurement of SYPRO Orange. **C** Thermal activation of PmAcy after 1-h (●, blue line) and 24-h incubation (□, green line) at various incubation temperatures and assayed according to the standard protocol at 30 °C. **D** Thermal stability of PmAcy after 1 h (●, blue line), 24 h (□, green line), and 4 days (▽, red line) incubation at various temperatures in 100 mM Tris–HCl pH 8.0, 150 mM NaCl, and 1 mM ZnCl_2_. All reactions were conducted in triplicate

The temperature dependency of the hydrolysis reaction rate was assayed in a range from 20 to 90 °C. The maximum activity was reached at 75 °C with 773 U/mg at pH 9.0 (Fig. 4A). To assess the thermal stability at these high temperatures, a thermal shift assay was performed. The enzyme sample was mixed with the fluorogenic dye Sypro orange. The fluorescence increases upon denaturation of the protein while allowing the dye to bind to newly exposed hydrophobic patches. Hence, heat-induced denaturation of the investigated enzyme can be observed in real time. Thermal denaturation starts at approximately 78 °C, and the melting point of PmAcy is 89 °C. Before the fluorescence signal increases due to denaturation of the enzyme, the signal decreases, starting from an extraordinary high level (Fig. 4B). This might be due to exposed hydrophobic patches of the recombinant enzyme and folding during heating (Boivin et al. 2013).

Motivated by these results, we investigated whether heating of the enzyme may increase its activity permanently (Fig. 4C). After 1-h incubation at various temperatures and thorough cooling of the enzyme samples, hydrolytic activity at 30 °C was measured. Indeed, an enzyme activation was observed upon heating. The strongest effect was observed at 80 °C with a threefold increase of activity. After 24-h incubation at the respective temperatures, a similar behavior was observed, although counteracted by thermal instability. To assess the stability of the heat-activated PmAcy, the enzyme was first heat-activated at 80 °C for 1 h, cooled down and then further incubated at various temperatures. After 1 h, 24 h and 4 days, the residual hydrolytic activities were measured (Fig. 4D). The enzyme was remarkably heat-stable. After 1 h at 80 °C, no significant loss in activity was found. At 70 °C, residual activities were 83% and 38% after 24 h and 4 days, respectively. At 50 °C, even 81% of activity was retained after 4 days. Additionally, stability after freezing the purified enzyme at -20° or -80 °C and subsequent thawing was investigated with and without the addition of 20% and 50% glycerol. No loss of activity was detected compared to the control kept at 4 °C.

### Substrate specificity of PmAcy in hydrolysis reactions and effect of metal ions

After purification of the enzyme without any bivalent metal ions added, no activity was retained. We found that 50 µM CaCl_2_, CuCl_2_, FeSO_4_, MgCl_2_, and NiCl_2_ could not restore activity, whereas the addition of ZnCl_2_, CoCl_2_, and MnCl_2_ reactivated PmAcy. The hydrolytic activities remained at the same level, when the metal ions were used at 100 µM, but were approximately halved when the enzyme was incubated with 10 µM metal ions. At concentrations of 1 µM hardly any activity was detected. Thus, the substrate specificity of PmAcy was investigated with various acylated L-amino acids in the presence of 50 µM ZnCl_2_, CoCl_2_, or MnCl_2_ (Table 1). Reactions were conducted at 15 mM substrate concentration in 100 mM Na-borate pH 9.0 at 50 °C to ensure solubilization of all substrates. L-amino acid released by PmAcy hydrolysis was quantified with the ninhydrin assay.

**Table 1:** Substrate specificity of PmAcy. Hydrolytic activity was measured at 15 mM substrate in 100 mM Na-borate pH 9.0. The reaction was conducted at 50 °C. Released amino acid were measured with the ninhydrin assay and activity was determined from the initial reaction velocity. The enzyme and Me^2+^ concentrations were 6–60 µg/mL and 50 µM, respectively. All reactions were conducted in triplicates

Substrate	Specific activity (U/mg)		
	ZnCl_2_	CoCl_2_	MnCl_2_
N-Lauroyl-L-alanine	184.4 ± 1.7	238.9 ± 4.6	63.9 ± 1.3
N-Acetyl-L-alanine	< 1	n.d	n.d
N-Benzoyl-L-alanine	0	n.d	n.d
N-Palmitoyl-L-alanine	60.4 ± 4.8	125.3 ± 7.9	58.6 ± 7.6
L-Phenylalanyl-L-alanine	0	n.d	n.d
N-Lauroyl-L-aspartic acid	0	0	0
N-Lauroyl-L-cysteine	0	0	0
N-Lauroyl-L-glutamic acid	0	0	0
N_α_-Lauroyl-L-glutamine	69.1 ± 3.3	105.8 ± 13.5	16.6 ± 1.2
N_α_-Capryloyl-L-glutamine	10.5 ± 0.4	22.8 ± 0.6	4.3 ± 0.3
N_α_-Palmitoyl-L-glutamine	38.8 ± 4.2	85.6 ± 1.1	28.2 ± 0.7
N-Lauroyl-glycine	123.9 ± 2.1	166.2 ± 5.4	23.6 ± 2.3
N-Lauroyl-L-isoleucine	148.9 ± 4.1	123.1 ± 1.6	39.5 ± 2.0
N-Lauroyl-L-leucine	32.3 ± 3.4	43.4 ± 3.4	47.0 ± 3.2
N-Lauroyl-L-methionine	177.8 ± 13.6	121.5 ± 1.7	55.3 ± 4.1
N-Lauroyl-L-phenylalanine	78.0 ± 3.7	80.6 ± 1.4	57.4 ± 4.1
L-Alanyl-L-phenylalanine	0	n.d	n.d
N-Lauroyl-L-serine	20.4 ± 4.0	31.7 ± 2.1	17.9 ± 2.2
N_α_-Lauroyl-L-tryptophan	4.9 ± 1.3	17.6 ± 0.1	2.1 ± 0.4
N-Lauroyl-L-tyrosine	63.5 ± 6.5	10.1 ± 0.9	30.3 ± 1.2
N-Lauroyl-L-valine	130.6 ± 2.3	168.7 ± 13.6	40.9 ± 1.8

The enzyme has a bias for lauric acid and non-polar and neutral L-amino acids (Table 1). With zinc, favored substrates were N-lauroyl-L-alanine, N-lauroyl-L-isoleucine, N-lauroyl-L-methionine, and N-lauroyl-L-valine at the chosen conditions. Regarding the fatty acid moiety, N-palmitoyl-L-alanine was hydrolyzed at a rate of 36% compared to N-lauroyl-L-alanine. On the other hand, N-acetyl-L-alanine and various other acetylated amino acids were barely hydrolyzed. Similarly, the activity against N-palmitoyl-L-glutamine and N-capryloyl-L-glutamine was 50% and 13% of N-lauroyl-L-glutamine, respectively, but no activity against N-acetyl-L-glutamine was measured. The aminoacylase also showed no dipeptidase activity against L-Ala-L-Phe and L-Phe-L-Ala. When the enzyme was incubated with 1 mM of bivalent cobalt ions, the substrate scope was altered. However, no additional substrates were accepted. The specific activity against N-lauroyl-L-alanine was altered from 184.4 U/mg with ZnCl_2_ to 239.0 U/mg with CoCl_2_ and to 63.9 U/mg with MnCl_2_. For N-lauroyl-L-phenylalanine, the differences were smaller (78.0 U/mg, 80.6 U/mg, and 57.4 U/mg, respectively). Remarkably, activity against N-palmitoyl-L-alanine and N-palmitoyl-L-glutamine was increased when cobalt instead of zinc was added to the enzyme (from 60.4 U/mg to 125.3 U/mg and from 38.8 U/mg to 85.6 U/mg, respectively). As the cobalt-activated enzyme also hydrolyzed N-capryloyl-L-glutamine faster, the specificity for the acyl moiety seems to be loosened. This trend was more pronounced when the enzyme was incubated with manganese, observing almost the same reaction velocity for N-lauroyl- and N-palmitoyl-L-alanine. However, since the activity was generally lower, the activity with manganese never exceeded the activity with zinc or cobalt for any substrate, except for N-lauroyl-L-leucine.

Kinetic values for the hydrolysis of N-lauroyl-L-alanine and N-lauroyl-L-phenylalanine at 30 °C were determined by measuring initial reaction rates at varying concentrations from 50 µM to 15 mM. The *K*_*M*_ and *V*_*max*_ values against N-lauroyl-L-alanine were found to be 1.95 mM and 55.4 U/mg, respectively. In contrast, for N-lauroyl-L-phenylalanine, the *K*_*M*_ value was 0.21 mM, and *V*_*max*_ was 25.1 U/mg (Fig. S3).

### Biocatalytic N-acyl-L-amino acid synthesis in aqueous media

**Fig. 5:**
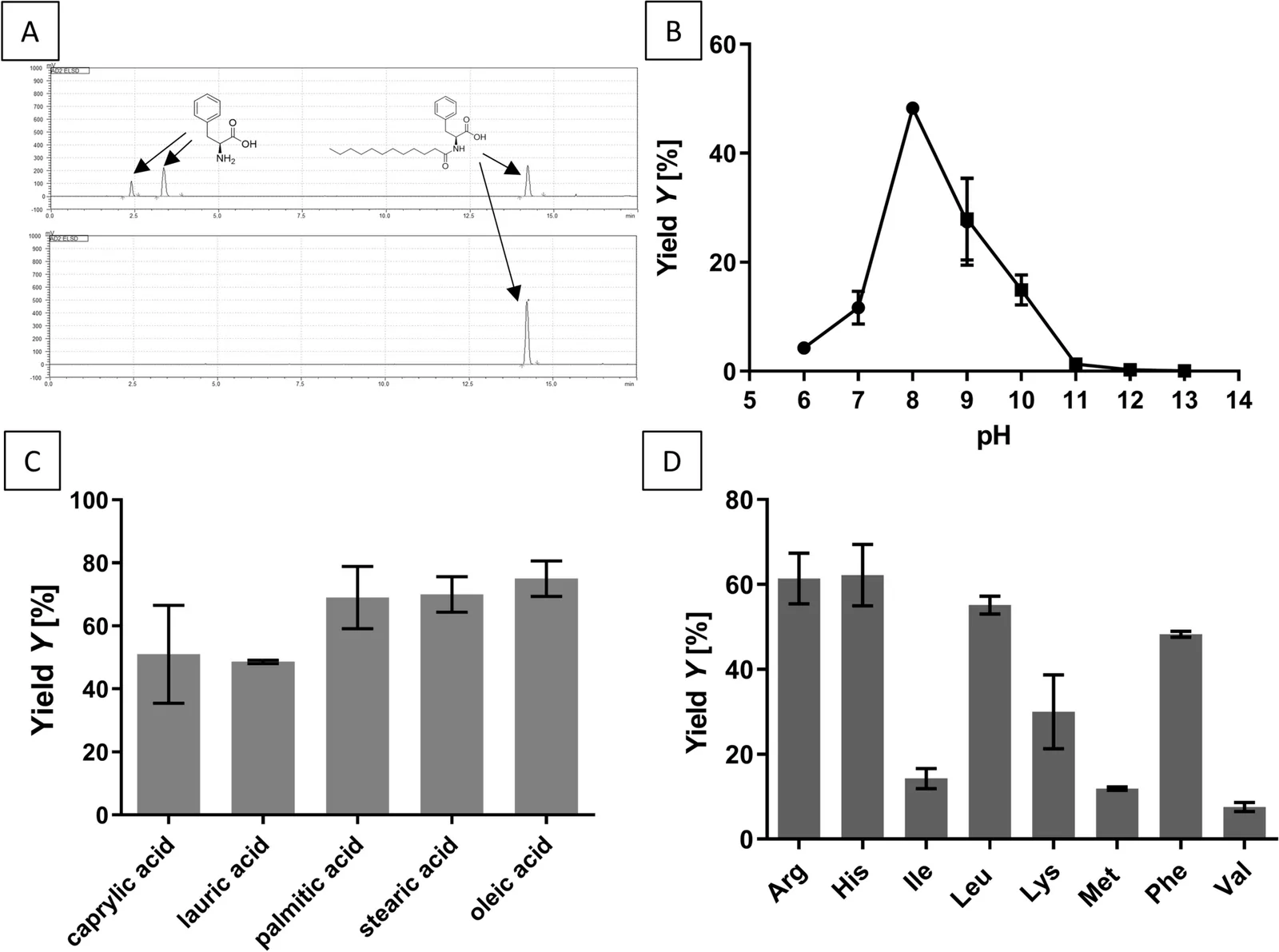
The synthetic potential of PmAcy for the formation of N-acyl-amino acids. Substrate concentrations were 200 mM amino acid and 100 mM fatty acid. **A** HPLC-ELSD chromatogram of the product N-lauroyl-L-phenylalanine synthesized with PmAcy after 24 h (upper picture) and after purification via acid precipitation (lower picture). **B** pH dependency of N-lauroyl-L-phenylalanine synthesis in 50 mM Tris buffer (●, pH 6.0–9.0) and 50 mM borate buffer (■, pH 10.0–13.0) at 50 °C for 24 h; all reactions were made in triplicates; **C** Synthesis of N-acyl-L-phenylalanine with different fatty acids (C8:0 = caprylic acid, C12:0 = lauric acid, C16:0 = palmitic acid, C18:0 = stearic acid, C18:1 = oleic acid); all transformations were performed in duplicates. **D** Synthesis of N-lauroyl amino acids with different amino acids (given as 3-letter code); all transformations were done in triplicates; fatty acid and amino acid variations were analyzed at 50 °C in 50 mM Tris buffer pH 8.0

The synthesis of N-acyl-L-amino acids was initially tested with L-alanine and lauric acid as substrates, since N-lauroyl-L-alanine proved to be the best substrate in the hydrolysis reaction. High substrate concentrations with 100 mM lauric acid and a two-fold stoichiometric excess of L-alanine (200 mM) were utilized. Despite being a favored substrate in hydrolysis, only trace amounts of N-lauroyl-L-alanine were detected in HPLC-ELSD analysis, and thus, synthesis reactions were repeated with L-phenylalanine under the same conditions. Formation of the acylated product was detected in HPLC-ELSD, which corresponded to the retention time of the chemically synthesized N-lauroyl-L-phenylalanine reference substance (Fig. 5A; HPLC ELSD chromatogram overlay). To verify the structure of the new product, it was enriched by acid precipitation according to the standard procedure employed in Schotten-Baumann synthesis and extracted (Fig. 5A). LC–MS analyses (Fig. S4) and NMR spectroscopy (Fig. S5, S6) unambiguously confirmed the formation of N-lauroyl-L-phenylalanine.

The synthesis potential of the enzyme was then tested in the pH range from 6.0 – 13.0 (Fig. 5B). The highest conversion for synthesis was observed at pH 8.0, whereas the highest initial activity of the enzyme was at pH 12.0 for the hydrolysis reaction. At pH 7.0 and below, the substrates were not completely soluble initially forming a cloudy mixture, whereas raising the pH above 10.0 seems to drastically lower the yield of the reaction. A pH of 8.0 was chosen for all further synthesis experiments. The chain length specificity of fatty acids was tested in the range of C8–C18 with L-phenylalanine as co-substrate (Fig. 5C). For all fatty acids of chain lengths C16 and C18, 10% (v/v) ethanol had to be added to facilitate sufficient solubilization of the hydrophobic substrate in the aqueous reaction system. The synthesis of medium- and long-chain N-acyl-L-phenylalanine showed good conversions between 50 and 75%. Higher conversions of 70–75% were obtained with the C16-C18 fatty acid substrates, while slightly lower conversions of 50–55% were obtained with C8 and C12 fatty acid substrates. The lowest transformation rate was observed with short chain caprylic acid and lauric acid.

Following these results, the conversion of all 20 natural L-amino acids was tested using lauric acid as co-substrate. As Fig. 5D shows, L-histidine, L-arginine, L-leucine, and L-phenylalanine showed the highest conversion rates with 62%, 61%, 55%, and 48% yield after 24 h reaction at 50 °C, respectively, while slightly lower yields between 7 and 30% yield were obtained with L-isoleucine, L-lysine, L-methionine, and L-valine. In the condensation reaction, the enzyme seems to favor highly hydrophobic and basic amino acid side chains, while other polar and weakly non-polar L-amino acids (L-alanine, L-asparagine, L-serine, L-threonine, L-tryptophan, and L-tyrosine) produced only traces of product (not shown). Acidic L-amino acids (L-aspartic acid, L-glutamic acid) as well as L-glutamine, L-cysteine, glycine, and L-proline were not accepted as substrates under the reaction conditions applied.

## Discussion

We described the cloning, expression, characterization, and the evaluation of the biocatalytic potential of the novel aminoacylase PmAcy. The successful expression in recombinant *E. coli* cells described in this study has not been achieved so far; several other aminoacylases were produced in the native host, or heterologous expression was not successful (Takakura and Asano 2019; Bourkaib et al. 2020a). The expression of a mycobacterial aminoacylase could be enhanced through the co-expression of GroEL/S in *E. coli* and *V. natriegens* (Haeger et al. 2023). We developed a protocol that enables soluble expression of the recombinant enzyme involving autoinduction with lactose and co-expression of the GroEL/S chaperonine.

The enzyme’s stability at alkaline pH values and high temperatures is extraordinary for aminoacylases and has only been described for the homologous aminoacylase from *Burkholderia* sp. (Takakura and Asano 2019). This distinctive trait is especially interesting for use in enzymatic synthesis of N-acyl-L-amino acids, because the disadvantage of other known aminoacylases is often their insufficient stability (Wardenga et al. 2010; Bourkaib et al. 2020b). In this context, we observed an enhanced activity of the purified enzyme after incubation at high temperatures. This permanent heat-activation has not yet been described in literature. As PmAcy is prone to the formation of multimers, this may hint at a temperature-dependent disintegration of enzyme aggregates.

The aminoacylase was active with the addition of Zn^2+^, Co^2+^, and Mn^2+^. The dependency of activity on divalent cations has been observed for other aminoacylases as well. The homologous aminoacylase from *Burkholderia* sp. strain LP5_18B was described to be zinc-dependent (Takakura and Asano 2019). Furthermore, several other aminoacylases were found to be activated by divalent cations, namely, zinc, cobalt, or nickel (Bourkaib et al. 2020a; Koreishi et al. 2009b; Natsch et al. 2003). Regarding hydrolysis of various N-acyl-L-amino acids, PmAcy preferred rather small, hydrophobic L-amino acids among the tested substrates. The best substrates were N-lauroyl-L-alanine, N-lauroyl-L-isoleucine, N-lauroyl-L-methionine and N-lauroyl-L-valine. Some polar, uncharged acylated amino acids, like N-lauroyl-L-serine, and N-acyl-L-glutamines were accepted to a lesser extent. However, N-lauroyl-L-cysteine and the charged N-lauroyl-L-glutamic acid and N-lauroyl-L-aspartic acid were not accepted at all. Hydrolysis of acylated L-histidine, L-arginine, and L-lysine could not be investigated during this study due to missing reference products or insolubility of the substrates.

The optimal pH for the enzymatic production of N-lauroyl-L-phenylalanine was pH 8.0. This pH value is much lower than the optimal pH for hydrolysis. In chemical synthesis with acyl chlorides, an alkaline pH is favorable for the synthesis of N-acyl-L-amino acids. With free fatty acids, which deprotonate in neutral or alkaline solutions, the optimal pH for synthesis can however be different in biocatalysis. The optimal pH for the reaction is also determined by the micro-environment of the enzyme’s active site and could therefore be unique for each enzyme. For the close homolog from *Burkholderia* sp. LP5_18B, the optimal pH for synthesis of N-lauroyl-L-phenylalanine was pH 9.0, despite having its hydrolytic optimum at pH 12.0. For SamAA, an aminoacylase from *S. ambofaciens*, slightly basic pH of 8.0 was best for maximal yield and pH 8.5 for highest initial velocity of the synthesis of N-undecylenoyl-L-phenylalanine (Bourkaib et al. 2020b). The optimal pH of porcine pAcy1 was around 7.5 in the synthesis of N-lauroyl-L-lysine, N-lauroyl-L-methionine, and N-lauroyl-L-glutamic acid (Wada et al. 2002).

Comparing various aminoacylases from literature, the activity against fatty acids of varying chain length differs. Often, there is a bias towards short-chain acyl residues, e.g., acetic acid, with promiscuous, lower activity to longer acyl chains (Koreishi et al. 2009b, 2005; Haeger et al. 2023). However, in case of the *Burkholderia* aminoacylase and penicillin V acylases, N-lauroyl-L-amino acids are hydrolyzed with high activity, whereas N-acetyl-L-amino acids are not accepted as substrates. The aminoacylase from *P. monticola* described in this study behaves like the latter case, barely hydrolyzing N-acetyl-L-amino acids and favoring N-lauroyl-L-amino acids in hydrolysis. In synthesis, PmAcy even favors long-chain stearic and oleic acids, thus rendering it a long-acyl chain aminoacylase both in hydrolysis and in synthesis. A discrepancy in hydrolytic and synthetic substrate scope is noticeable, most strikingly as N-lauroyl-L-alanine is the favored substrate in hydrolysis but is barely synthesized. A similar behavior has been described for the *Burkholderia* enzyme (Takakura and Asano 2019). Furthermore, PmAcy hydrolyzed N-lauroyl-L-isoleucine faster than N-lauroyl-L-leucine; however, synthesized N-lauroyl-L-leucine is more efficiently. Hence, biocatalytic substrate scope might not only be dependent on substrate binding to the enzyme’s active site but also by thermodynamic equilibrium and product solubility.

In summary, we identified a novel aminoacylase from *P. monticola* that is capable of long-chain N-acyl-L-amino acid synthesis. Synthesis of N-acyl-L-amino acids was possible in aqueous conditions in high yield, which is ecologically advantageous to the industrially applied Schotten-Baumann reaction and eventually more economical. Thus, we anticipate the described aminoacylase to be highly attractive for biotechnological applications.

## Supplementary Information

Below is the link to the electronic supplementary material.

Supplementary file1 (PDF 458 KB)

## Data Availability

Data are available on request.
