# Supplementary material for: Novel recombinant aminoacylase from Paraburkholderia monticola capable of N-acyl-amino acid synthesis
Source: Appl Microbiol Biotechnol. 2024 Jan 10;108:93. doi: 10.1007/s00253-023-12868-8 (PMC10781821; doi:10.1007/s00253-023-12868-8)
Supplement: Supplementary file 1 [file 253_2023_12868_MOESM1_ESM.pdf]

Supplementary materials

Journal name:

Applied Microbiology and Biotechnology

Title:

Novel recombinant aminoacylase from *Paraburkholderia monticola* capable of N-acyl-amino acid synthesis

Author's names:

Gerrit Haeger[a], Tristan Jolmes[b], Sven Oyen[a], Karl-Erich Jaeger[c,d], Johannes Bongaerts[a], Ulrich Schörken[b], and Petra Siegert[a]

Addresses:

[a]Institute of Nano- and Biotechnologies, Aachen University of Applied Sciences, 52428 Jülich, Germany

[b]TH Köln University of Applied Sciences - Leverkusen Campus, Faculty of Applied Natural Sciences, Leverkusen, Germany

[c]Institute of Molecular Enzyme Technology, Heinrich Heine University Düsseldorf, 52425 Jülich, Germany

[d]Institute of Bio- and Geosciences IBG-1: Biotechnology, Forschungszentrum Jülich GmbH, 52425 Jülich, Germany

Author for correspondence:

Petra Siegert; Heinrich-Mussmann-str. 1, 52428, Jülich, Germany. E-mail address: siegert@fh-aachen.de (P. Siegert), Tel.: +49 241 6009 53124

Supplementary

Protein sequence of PmAcy (N-terminal Strep-tag and linker underlined):

MWSHPQFEKSGMLSLVLPGLSQAQSTLPPAPPAKPVLFTNFRLFDGKSMTLRDGLYMVVEGNSISQLG
QGQPASVEGKTLVDCGGKVMMPGLIDMHWHALLAALPIQVILQSDIAFVHLAASAEAERTLLRGFTTI
RDAGGPSFALKQAIDSGMISGPRIYPSGAMITTTGGHGDFRPLTDLPRTSSQVTQGERDGGFAIADTE
DEMRVRVREQFIQGATQIKLVGSGGVSTPRSPLDMLTFTEKQLRAAVETAADWGTYVLSHAYTPEAVQ
RSVAAGAQCIEHGHLMDDKTAALMAKNGTWLSTQPFISEEDVGPLAPQSREKFLEVVAGTDNAFRLAR
KHGIKVAFGTDLLFSQAIATRQGTMLTHMKRWYSPAEALGMATGTNGQLLALTGKRNPYPRRLGVLEE
GAYADLLLVDGNPLENLDLIANPEQNLRIVMKDGKFYKNTLKA

Codon -optimized sequence with N-terminal strep-tag (as ordered from GeneArt, Thermo Fisher); GenBank OR188138:

ATGTGGTCACATCCGCAGTTTGAAAAAAGCGGTATGCTGAGCCTGGTTCTGCCTGGTCTGAG
CCAGGCACAGAGCACCCTGCCTCCGGCACCGCCTGCAAAACCGGTTCTGTTTACCAATTTTC
GTCTGTTTGATGGCAAAAGCATGACCCTGCGTGATGGTCTGTATATGGTTGTTGAAGGTAAT
AGCATTAGCCAGTTAGGTCAGGGTCAGCCTGCAAGCGTGGAAGGTAAAACCCTGGTTGATTG
TGGTGGTAAAGTTATGATGCCAGGTCTGATTGATATGCATTGGCATGCACTGCTGGCAGCAC
TGCCGATTCAGGTTATTCTGCAGAGCGATATTGCATTTGTTCATCTGGCAGCAAGCGCAGAA
GCAGAACGTACCCTGCTGCGTGGTTTTACCACAATTCGTGATGCCGGTGGTCCGAGCTTTGC
ACTGAAACAGGCAATTGATAGCGGTATGATTAGCGGTCCGCGTATTTATCCGAGCGGTGCAA
TGATTACCACAACCGGTGGTCATGGTGATTTTCGTCCGCTGACCGATCTGCCTCGTACCAGC
AGCCAGGTTACCCAGGGTGAACGTGATGGTGGTTTTGCAATTGCCGATACCGAAGATGAAAT
GCGTGTTCGTGTGCGTGAACAGTTTATTCAGGGTGCAACACAGATTAAACTGGTTGGTAGCG
GTGGTGTTAGCACACCGCGTAGTCCGCTGGATATGCTGACCTTTACCGAAAAACAGCTGCGT
GCAGCAGTTGAAACCGCAGCAGATTGGGGCACCTATGTTCTGAGCCATGCATATACACCGGA
AGCAGTTCAGCGTAGCGTTGCAGCCGGTGCACAGTGTATTGAACATGGTCATCTGATGGATG
ATAAAACAGCAGCACTGATGGCCAAAAATGGCACCTGGCTGAGCACCCAGCCGTTTATTAGC
GAAGAAGATGTTGGTCCGCTGGCACCGCAGAGCCGTGAAAAATTTCTGGAAGTTGTTGCAGG
CACCGATAATGCATTTCGTCTGGCACGTAAACATGGTATTAAAGTTGCATTTGGTACGGATC
TGCTGTTTAGCCAGGCCATTGCAACCCGTCAGGGCACCATGCTGACCCATATGAAACGTTGG
TATAGCCCTGCCGAAGCATTAGGTATGGCAACCGGCACCAATGGTCAGCTGCTGGCACTGAC
CGGTAAACGTAATCCGTATCCGCGTCGTCTGGGTGTTCTGGAAGAAGGTGCATACGCCGATT
TACTGCTGGTTGATGGTAACCCGCTGGAAAATCTGGATCTGATTGCAAATCCGGAACAGAAT
CTGCGTATTGTGATGAAAGATGGCAAGTTCTATAAGAACACCCTGAAAGCA**AGCGGTTGGAG**
CCATCCTCAGTTCGAGAAATGA

Primer sequences for cloning:

Table S1: Primer sequences used for amplification and cloning of *pmAcy Ntag*. BsaI-sites are highlighted in bold font.

No.	Name	Sequence 5’->3’
P1	NTag for:	**GGTCTC**CCATGTGGTCACATCCGCAGTTTGAAAAAAG
P2	noTag for:	**GGTCTC**CCATGCTGAGCCTGGTTCTGCC
P3	CTag rev:	**GGTCTC**TCTCATTTCTCGAACTGAGGATGGCTC
P4	noTag rev:	**GGTCTC**TCTCATGCTTTCAGGGTGTTCTTATAGAACTTGC

MALDI-TOF analysis:

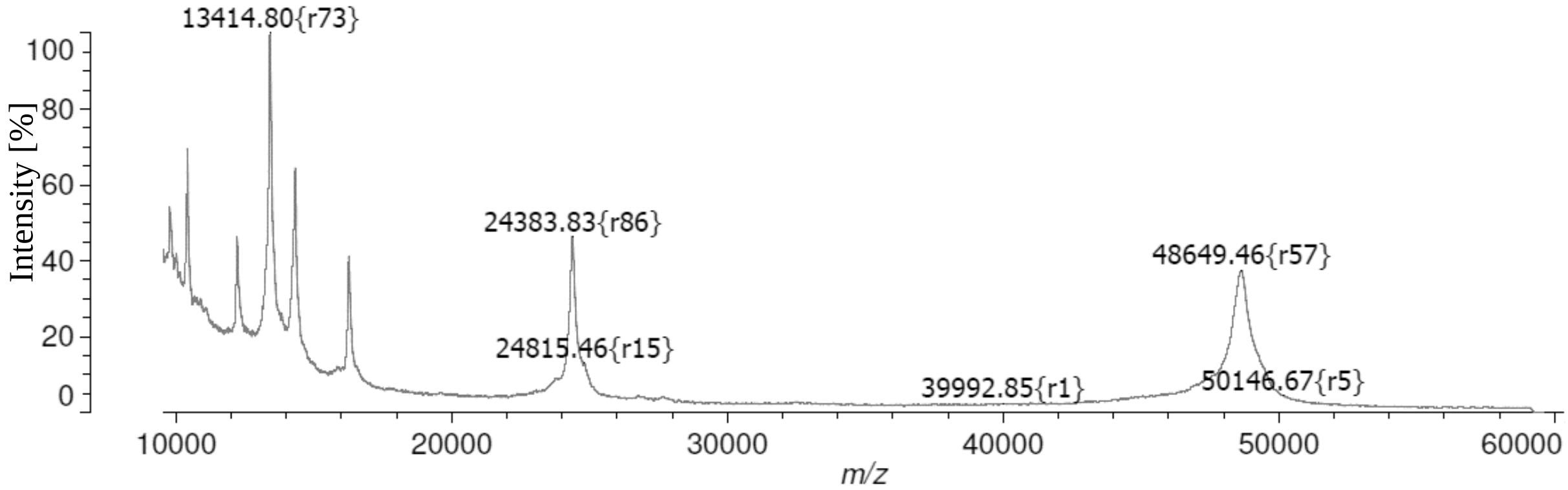

Figure S1: MALDI-TOF mass spectrum of PmAcy NTag (present in 100 mM Tris-HCl pH 8.0, 150 mM NaCl, 1 mM $ZnCl_2$); The relative intensity is plotted against the m/z-values. The numbers above the peaks indicate the measured m/z-value.

Native PAGE:

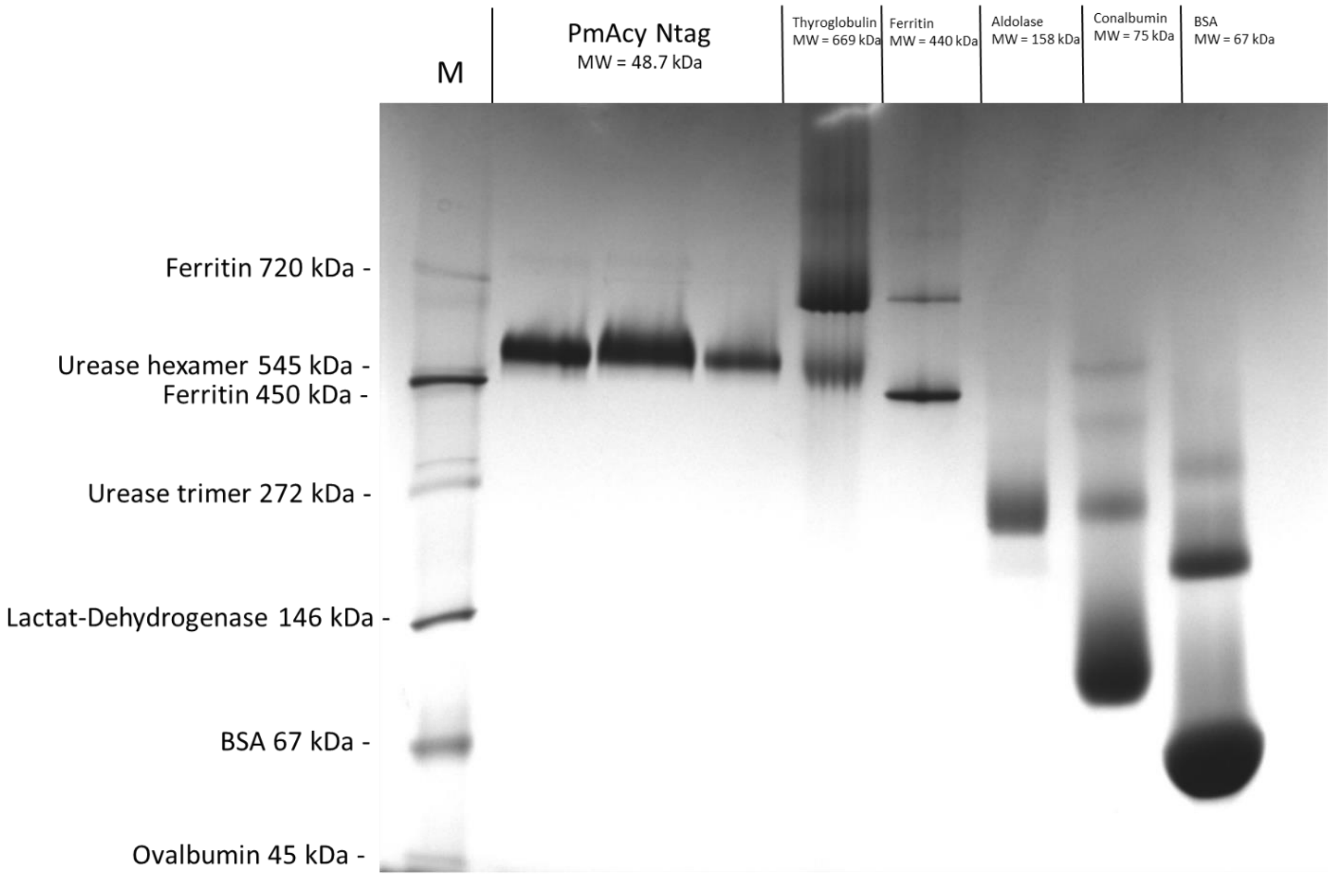

Figure S2: Blue native PAGE of PmAcy NTag and reference proteins. Lane 1: SERVA Native Marker, Liquid Mix for BN/CN (Cat.No. 39219.01); Lane 2-4: PmAcy NTag (three independent preparations); Lane 5: thyroglobulin, MW = 669 kDa; Lane 6: ferritin, MW = 440 kDa; Lane 7: aldolase, MW = 158 kDa; Lane 8: conalbumin, MW = 75 kDa; Lane 9: BSA, 67 kDa. Reference proteins from lane 5-8: Proteins from gel filtration calibration kit (High molecular weight, GE healthcare), BSA was from PanReac AppliChem).

Michaelis-Menten plot:

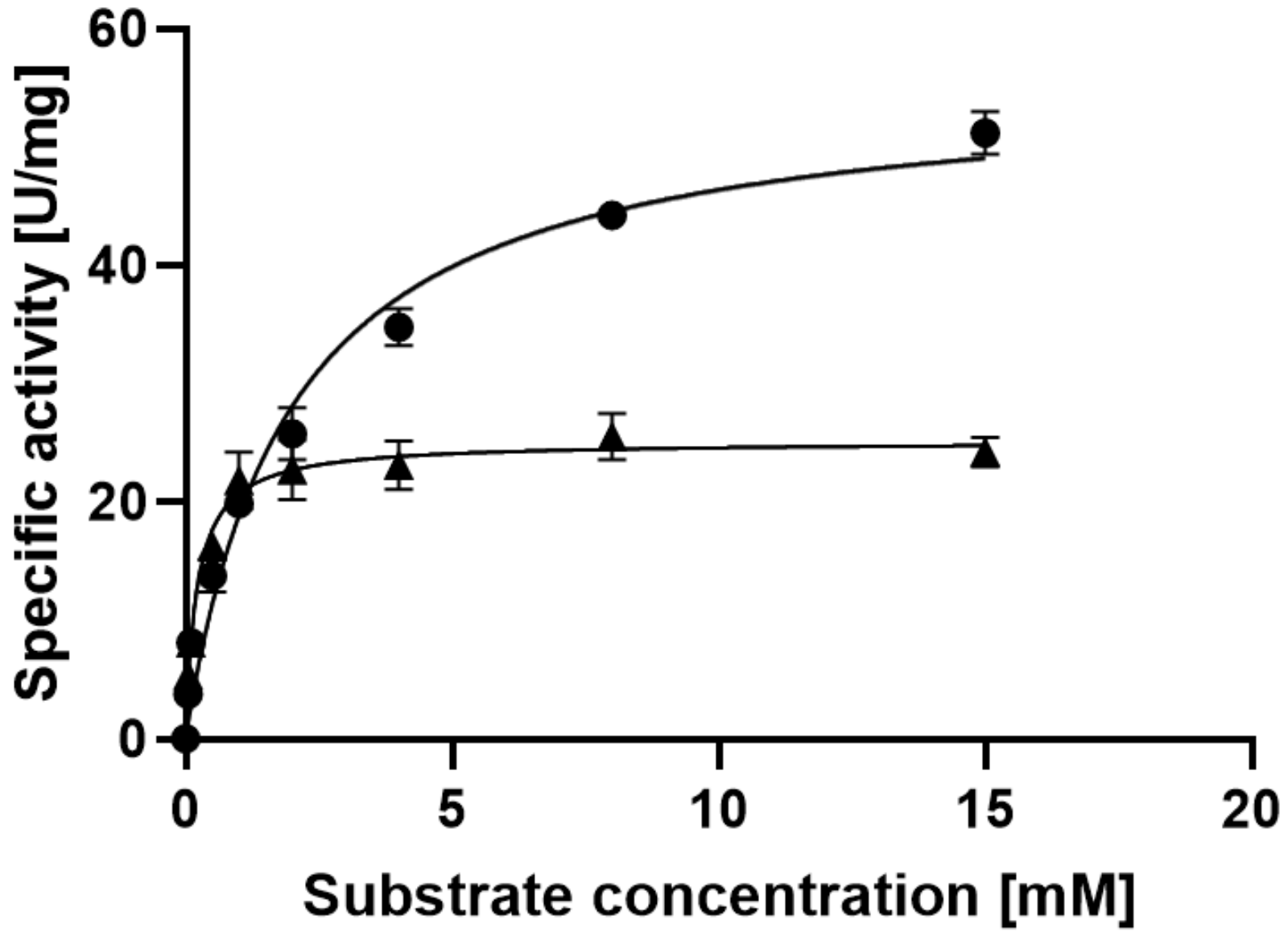

Figure S3: Michaelis-Menten hydrolysis kinetics of PmAcy NTag against lauroyl-alanine (●) and lauroyl-phenylalanine (▲). Non-linear fit to generate Michaelis-Menten plot was conducted with GraphPad Prism 8.

Analytical Data:
N-lauroyl-phenylalanine

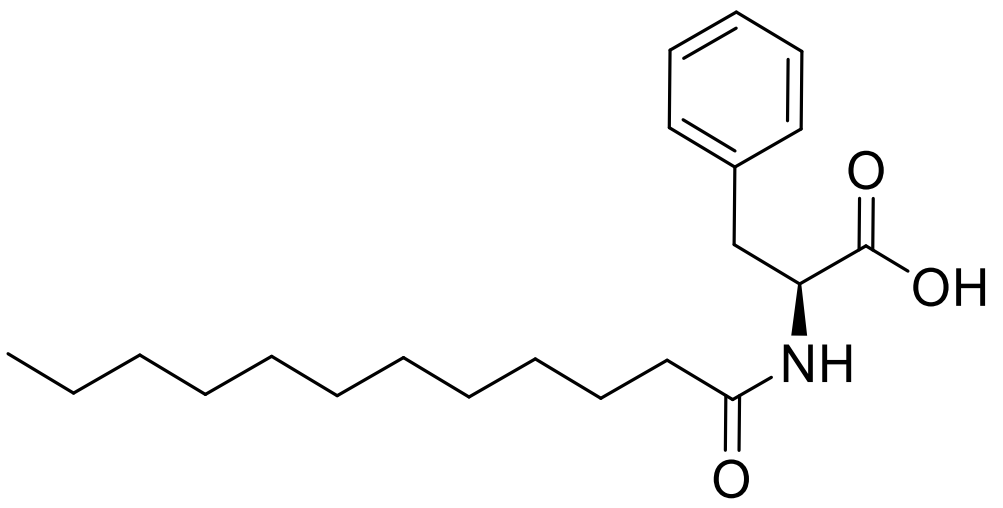

LC-MS: (EI, 70 eV): m/z [%] = 695 (2M+H, 80), 411 (M+ACN+Na, 10), 389 (M+ACN+H, 100), 348 (M+H, 100).
^{1}H-NMR ($CDCl_3$, 400 MHz) δ = 0.88 (t, ^{3}J = 7 Hz, 3H), 1.19 – 1.36 (m, 17 H), 1.48 – 1.59 (m, 2 H), 2.182 (dt, ^{2}J = 3.2 Hz, ^{3}J = 7.2 Hz, 2 H), 3.09 – 3.28 (m, 2 H), 4.88 (q, ^{3}J = 5.9 Hz, 1 H), 6.09 (d, ^{3}J = 7.5 Hz, 1 H), 7.13 – 7.18 (m, 2 H), 7.22 – 7.33 (m, 3 H).
^{13}C-NMR ($CDCl_3$, 100 MHz) δ = 14.14, 22.70, 25.59, 29.16, 29.32, 29.35, 29.48, 29.63, 29.93, 36.44, 37.27, 53.14, 127.22, 128.64, 129.38, 135.69, 174.14, 174.62.

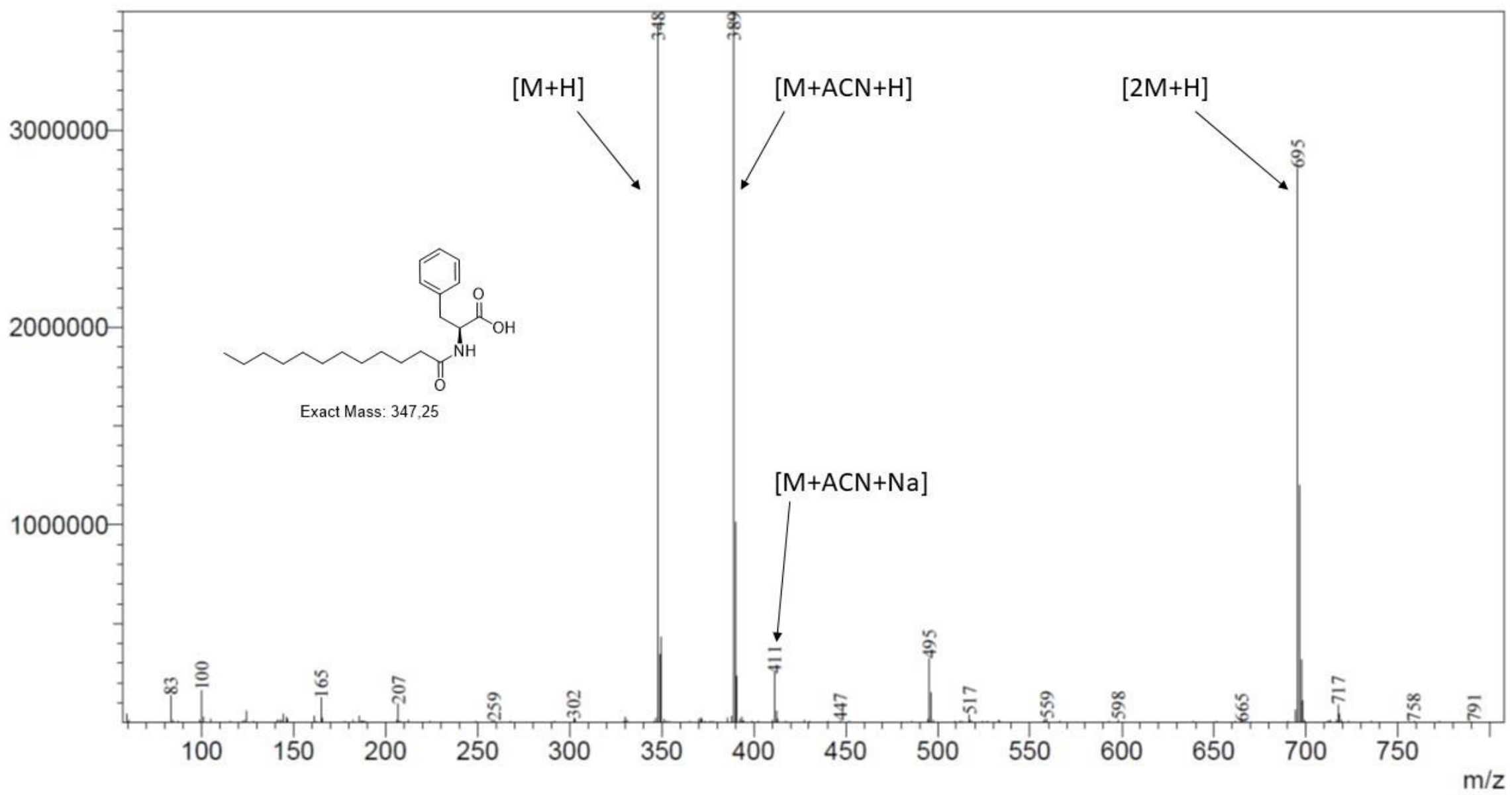

Figure S4: Mass-spectrum of N-lauroyl-phenylalanine, produced with PmAcy, isolated by acidic precipitation with 5 N HCl.

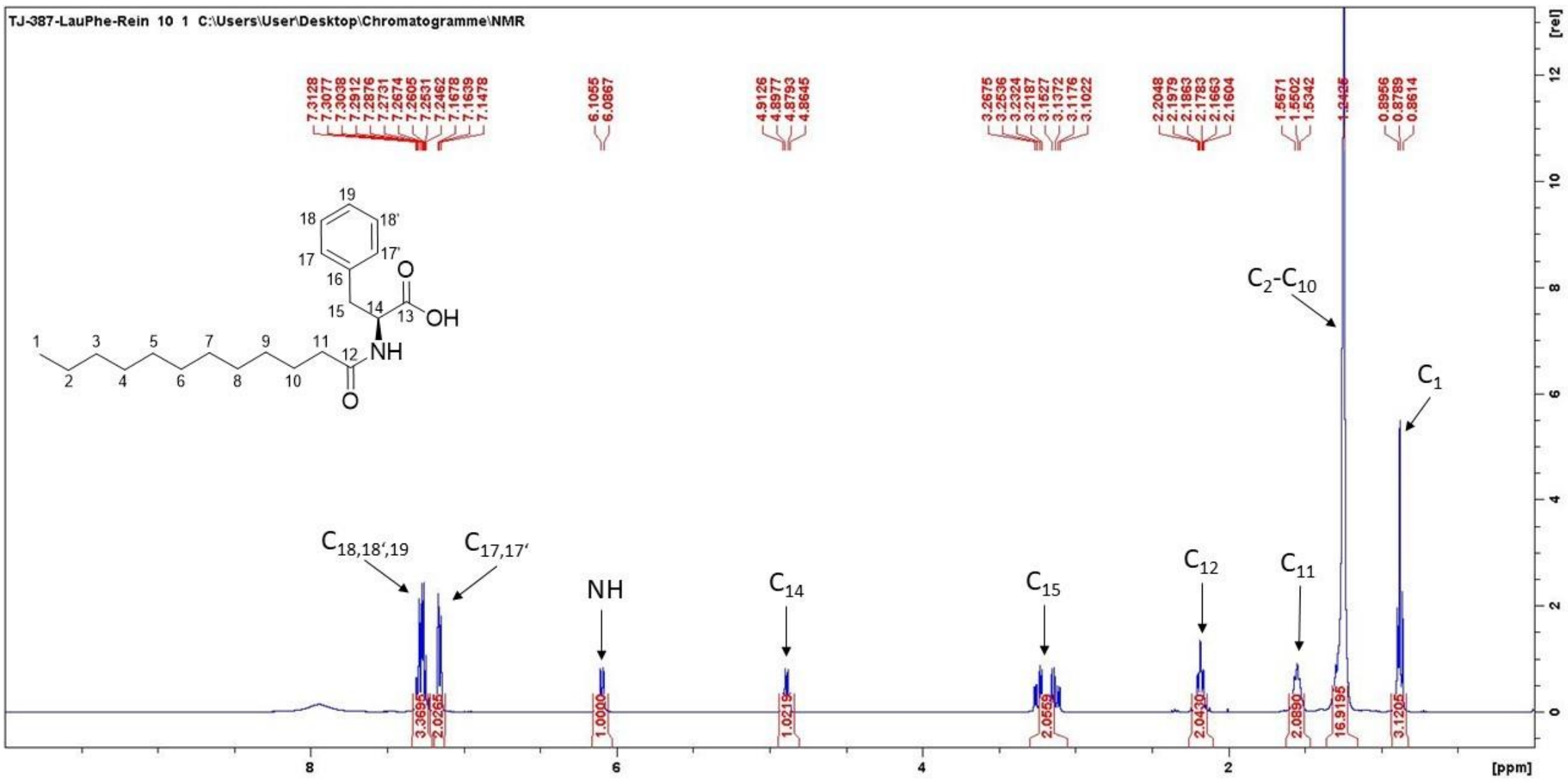

Figure S5: ^{1}H-NMR-spectrum of N-lauroyl-phenylalanine, produced with PmAcy, isolated by acidic precipitation with 5 N HCl.

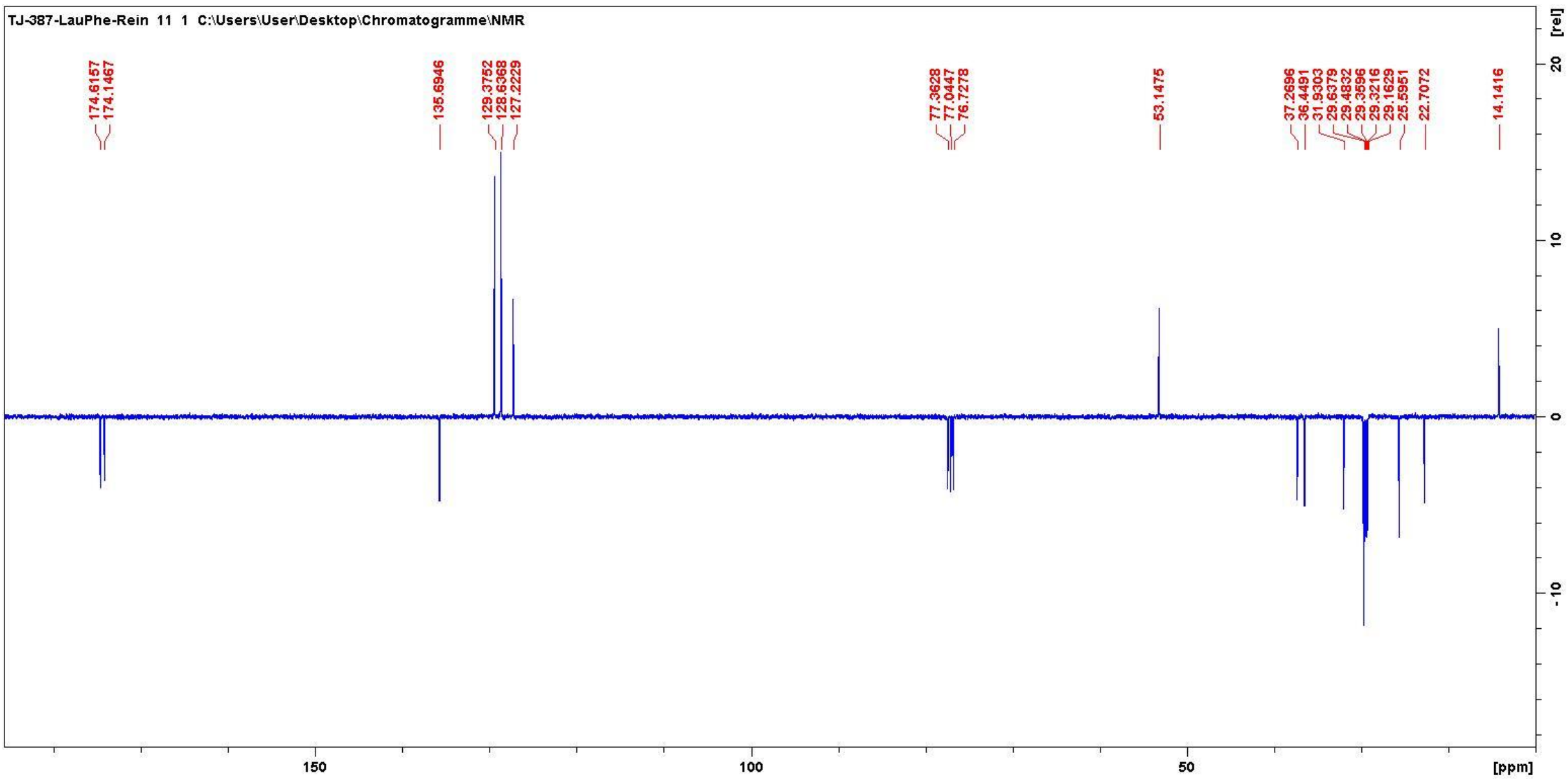

Figure S6: ^{13}C-NMR-spectrum of N-lauroyl-phenylalanine, produced with PmAcy, isolated by acidic precipitation with 5 N HCl.
